# Editorial

**Published:** 1844-06-15

**Authors:** 


THE MEDICAL EXAMINER.

PHILADELPHIA, JUNE 15, 1844.

### HEALTH OF PHILADELPHIA.

The health of the city, and small number of deaths as exhibited in the bills of mortality, is the daily subject of remark among all classes of the inhabitants at the present time. Struck by this common observation, we were induced to call at the Health Office for details. The statement for the last year, we found, was not yet printed, and so we merely referred to the books for a comparison of the reports of the present season with the corresponding period of last year. It appears that during the three months of March, April and May, 1843, the average weekly number of deaths was one hundred and fourteen and five-tenths. The same months of the present year give an average of one hundred and seven, or seven less per week. Last year was confessedly one of the most healthy within the recollection of the older physicians: since then, we have had a large accession to the number of our population, and yet the mortality is less. This is to be accounted for from the absence of all epidemic influences of either a general or local character, during this period, and from the circumstances peculiar to the place. Philadelphia enjoys, in a high degree, those natural advantages of climate and soil, which are necessary to render a place salubrious. Art has likewise contributed materially to the same end. By a well devised system of works, the supply of pure water is abundant, not only for all household purposes, but also to allow of its free use in washing out the narrow streets and water courses as often as cleanliness may require. The streets are wide, and being laid out at right angles to each other, permit a free circulation of air from whatever point of the compass the wind may blow. These circumstances obviate, in a great measure, the inconveniences ordinarily experienced by a congregated population. The abundant supply of excellent provisions, too, afforded by the surrounding country, contributes much to the healthfulness, as it undoubtedly does to the comfort and economy of the inhabitants. Such, indeed, is the productive character of the country immediately surrounding the city of Philadelphia, that large quantities of butter, fresh meat, fruit, and green vegetables, are daily bought in her markets for the supply of neighboring cities, particularly New York. Add to all these the boundless supply of her pure Anthracite coal, always to be had at a price within the means of the poorest of her labouring population, and no where, probably, can we find a concurrence of circumstances more favourable to human health.

---

### TRANSACTIONS OF THE NEW YORK STATE MEDICAL SOCIETY.

This is a valuable publication, containing the proceedings of the New York State Medical Society, at its annual meeting in February last. The papers read on the occasion, were:

1. Annual Address by the President, Dr. White, on Insanity.
2. Observations on Equivocal Generation, by Dr. Blatchford.
3. Medico-Legal Observations, by Dr. Willard.
4. On Poisoning by Arsenic, by Dr. Davis.
5. On Deaths from Poisoning, in the city and county of New York, by Dr. J. B. Beck.

These essays occupy seventy-two pages of the pamphlet, and are valuable contributions to medical science. The *Appendix* covers seventy-eight pages, and embraces a variety of matters, but chiefly reports, speeches, &c., in relation to medical reform and legal enactments for the prevention and suppression of Quackery. The physicians of that State, in concurrence with many of its best statesmen, have been battling for years with the many-headed monster, and seem to have very nearly come to the conclusion that the task is a hopeless one. This, indeed, is the experience in all countries where the government is directly responsible to public opinion; and even under the more despotic, the evil prevails, less openly, perhaps, but very much to the same extent. This is well explained in the following extract from the report of a committee of the Monroe County Medical Society:

"The object of legislation is the public good. Medical law may be said to have a twofold object; first, the protection of the community against imposition, where health and life are involved; second, the improvement of medical science; both aiming at and resulting in the public good. But law is the expression of the public will, without which it can neither be enacted, sustained, nor executed. The written statute is, therefore, a dead letter, whenever the public mind is arrayed against it. And this is pre-eminently the case with regard to the medical law of this state, at this time. Empiricism is every where rife, and was never more arrogant, and 'the people love to have it so.' That restless, agitating, agrarian spirit, that would always be levelling down, has so long kept up a 'hue and cry against calomel and the lancet,' that the prejudice of the community is excited against, and their confidence in the medical profession greatly impaired—and no law could be enforced against the empiric, or nostrum vender. Every attempt of the kind would only create a deeper sympathy in their favor, and raise a storm of higher indignation against the profession. This spirit cannot be controlled by arbitrary legislation. It only makes it, like compressed steam, the more elastic and more certain to explode. It is, by seeming to yield, and giving it space, that it becomes quiescent and harmless. A repeal of all penalties and disabilities, would take away from the quack his strong ground for appeal to the sympathies of the people."

The same intelligent committee, addressed a circular to some thirty individuals, (physicians,) residing in the different states, propounding the following queries:

"1. Is there any law in your state regulating the practice of physic and surgery? and if so, what is it?

"2. Do the laws of your state prohibit quackery, in any or all of its forms of Thompsonian, botanic, &c.?

"3. If any law of your state, imposing penalties or disabilities upon the quack has ever existed, has it ever been repealed or abolished? and if so, what influence has such abolishment had upon the increase or decrease of quackery?"

The opinions of the gentlemen addressed, are somewhat varient as it regards the effect of legal enactments upon the interests of the profession and of the community.

The following facts, however, are derived from their responses:

In New Hampshire, Rhode Island, Pennsylvania, Virginia, North Carolina, Kentucky, Tennessee and Missouri, no laws have ever been enacted for the regulation of the practice of medicine.

In the states of Maine, Vermont, Massachusetts, Connecticut, Maryland, South Carolina, Alabama, Mississippi, Indiana and Ohio, the laws which formerly existed on the subject, have been repealed, in some instances, "in accordance with the wishes" of the profession, as being in their opinion, "adverse to their interests."

In New Jersey, a law exists for the suppression of quackery, but from the array of popular opinion against it, its provisions are little regarded.

By a provision in the law enacted by the legislature of Georgia, in 1839, an exception is made in favor of Thompsonians, &c., by which the force of the law is entirely nullified.

In Louisiana and New York alone, are there any efficient laws on the subject; and in the latter state, such appears to be the force of popular feeling and the weakness of the statutory provisions, that many of the distinguished members of the medical profession believe that the existing laws on the subject, really promote charlatanry, by exciting the sympathy of the people in behalf of such pretenders. The committee of Monroe county very properly remark: "That the important, if not the only remedy against quackery, is medical reform, by which a higher standard of medical education shall be secured." Adopting the language of one of their correspondents, they observe: "We have too many schools of medicine that grind out doctors for the mere profit of it. As long as our Medical Colleges are mere machines for manufacturing M. D.'s according to the demand, the passage of laws by the States, although they may do something towards the suppression of quackery, can never wholly accomplish so desirable an end."

On this passage, we will only remark, that so long as the different State Legislatures continue to *multiply* Medical Colleges with the power of conferring degrees, without regard to the fitness of the place or the requirements of the community, so long will the evil continue. The only corrective consists in the individual example and efforts of the members of the profession. Let them, by continual rubbing, keep themselves bright—in other words, purchase the best and latest publications, and read them; send their pupils to colleges where something is to be acquired beside a diploma—let this be done, and the people will soon learn to distinguish between *physicians* and *quacks*.

